# Extraoral surgical removal of an ectopic impacted third molar of the mandible. Report of a case

**DOI:** 10.4317/jced.56602

**Published:** 2020-06-01

**Authors:** Lampros Goutzanis, Chara Chatzichalepli, Dimitrios Avgoustidis, Panagiotis Papadopoulos, Catherine Donta

**Affiliations:** 1 DDS, MD, MSc, PhD(Dent), PhD(Med), Assistant professor of Oral and Maxillofacial Surgery – Dental School, National and Kapodistrian University of Athens, Greece; 2 DDS, MSc, Postgrad in Dentoalveolar Surgery – Dental School, National and Kapodistrian University of Athens, Greece; 3 MD, DDS, Oral and Maxillofacial Surgeon – Private Practice, Athens, Greece; 4 MD, DDS, Oral and Maxillofacial Surgeon – Dental School, National and Kapodistrian University of Athens, Greece; 5 DDS, PhD, Associate Professor – Dental School, National and Kapodistrian University of Athens, Greece

## Abstract

Intraoral approach for the removal of impacted third molars represents a common surgical procedure for the specialized clinician. However, in some cases such as ectopic third molars, extraoral surgical removal seems to be inevitable. We present a step by step case of a 56 year old woman with an ectopic third molar of the lower jaw along with a cystic lesion, which were surgically removed by a submandibular approach. Postoperative clinical course was uneventful and there were no signs of facial nerve paresis. In such cases, appropriate preoperative planning must be made based on careful study of radiographic imaging and clinical examination. The more conservative technique that would minimize adjacent anatomic structures risk should be the surgical technique of choice.

** Key words:**Ectopic third molar, mandible, cyst, extraoral approach.

## Introduction

A tooth is considered as “ectopic” when it is located in an unusual site, distant from its natural anatomical position (1). Even though ectopic eruption limited within the dentoalveolar structures is not rare, the dislocation of a tooth in distant sites is infrequent (2,3). Impaction of third molars is found in 20-30% of the general population, with a predilection for women, however only a few cases of ectopic impacted third molars have been reported in the literature so far (1,4).

Ectopic third molars are more commonly found in the following anatomical areas: maxillary sinus, palate, mandibular condyle, coronoid process, mandibular ramus, angle and lower border of the mandible, the orbit, nasal cavity and surrounding soft tissues (2-6).

The etiology of the ectopic third molars has not yet been clarified. A variety of reasons could explain this displacement: Eruption, which is a natural process of tooth migration from an intraosseous to a functional site, whenever is aborted, could result in an ectopic impaction (4). Trauma in the region of eruption and ectopic formation of tooth germs has also been proposed as a possible mechanism (4). Additionally, contiguity with cystic or neoplastic lesions may lead to an altered location of the tooth (1-3,5).

Usually ectopic third molars are randomly found in panoramic radiographs during routine clinical examinations, as they remain asymptomatic for years (1,7). In some cases they may cause severe episodes of pain, swelling or trismus leading the patient to the clinician, where the diagnosis of an ectopic molar is placed in combination with a cystic or neoplastic lesion (8,9).

Current paper presents an interesting case of an ectopic impacted third molar in the lower border of the right mandible, associated with a cystic lesion, which was removed using an extraoral surgical approach. Main aspects such as diagnosis, pathology and treatment are extensively discussed for a comprehensive presentation of this subject.

## Case Report

**Figure 1:**
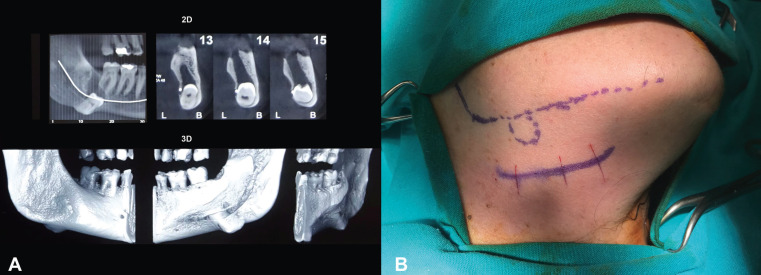
A) Selected 2D and 3D views from patient’s CBCT demonstrating that the impaction is mainly affecting the lingual plate of the lower border of the right mandibular body. B) Careful planning of the surgical procedure intraoperatively. The dotted straight line represents the lower border of the right mandible. The continuous straight line represents the skin incision (2cm below the lower border of the right mandible). The dotted circle represents the location of the impacted tooth.

A fifty-six-year old female referred to our clinic, reporting pain in the right side of her face and difficulties in mouth opening. She also reported that she was aware of a protrusion concerning the lower border of the right mandibular area for more than 10 years. Patient’s medical history did not include any concomitant diseases. Preliminary extraoral clinical examination showed a large painful inflammatory swelling located in the lower border of the right mandible. Panoramic x-ray revealed a deep impaction of an ectopic third molar in that area in combination with a radiolucent lesion. The cone beam computed tomography (CBCT) demonstrated that the impaction was mainly affecting the lingual plate of the lower border of the mandible (Fig. 1A).

Due to clinical signs implying inflammation, the decision to remove the tooth along with the cystic lesion was taken. After initial prescription of antibiotic treatment for the reminiscence of inflammatory symptoms, the patient underwent surgery.

**Figure 2:**
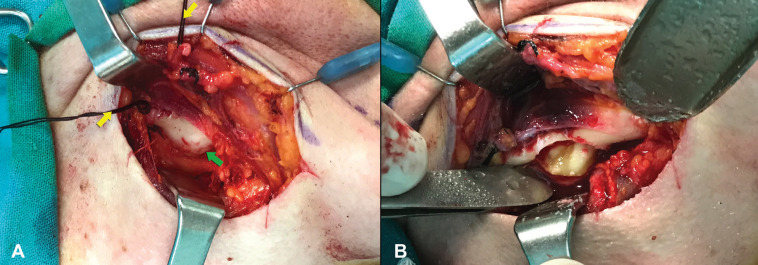
A) The surgical site after raising the periosteum of the mandibular body. The two yellow arrows point to the ligatures of the facial artery and vein which are used to retract the facial nerve from the surgical site. The green arrow points towards the protrusion of the impaction from the mandible. B) After osteotomy of the buccal plate covering the impaction site, the impacted third molar and the cyst are revealed.

**Figure 3:**
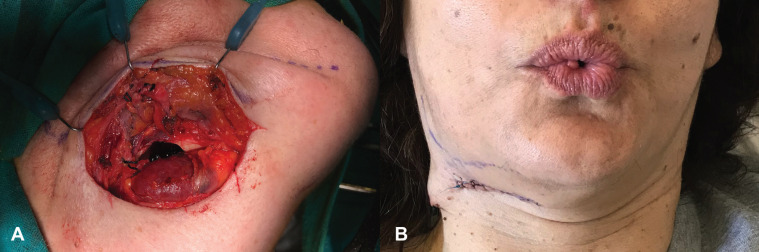
A) View of the surgical site immediately after removal of the impacted tooth and the cyst. B) No clinical signs of paresis or paralysis of the right marginal mandibular branch of the facial nerve, two days after surgical procedure.

Extraoral approach under general anesthesia was the surgical technique of choice. After careful preparation of the surgical site (Fig. 1B), a submandibular skin incision, nearly 2cm below the lower border of the right mandibular body was performed and platysma muscle was incised subsequently. The marginal mandibular branch of the facial nerve (VII) was identified immediately under the superficial layer of the deep cervical fascia and was retracted away from the surgical site after ligation of the facial vein and artery. The pterygomasseteric sling was then incised and incision of the periosteum covering the lower border of the right mandible followed. The impaction site could then be reached by carefully raising the periosteum from the underlying mandibular bone (Fig. 2A). Using a low-speed rotary instrument under sterile saline solution, osteotomy was performed revealing the impacted tooth (Fig. 2B). Immediately after, the impacted third molar was removed along with the cystic lesion (Fig. 3A). The inferior alveolar nerve was not exposed in the surgical site. The surgical trauma was thoroughly irrigated with antiseptic and saline solutions, consequently a surgical drainage was placed and soft tissues were closed in sequential layers.

The postoperative course was uneventful and clinical examination revealed no signs of paresis or paralysis of the marginal mandibular branch of the facial nerve (Fig. 3B). Pathology report confirmed that the radiolucent lesion was a dentigerous cyst.

## Discussion

Very few cases of ectopic mandibular third molars have been reported in literature so far (8,9). However, ectopic third molars constitute an interesting subject in terms of etiology of appearance, dislocation sites, clinical and radiographic features, as long as their contiguity with cystic lesions or tumors and ultimately treatment choices.

The etiology of ectopic tooth impaction has yet to be clarified. Several theories have been proposed such as alterations during odontogenesis and trauma to a developing tooth germ, pathologic and iatrogenic conditions or even crowding (1-6). More specific, a mandibular third molar may be dislocated at a greater distance from its habitual site due to aborted eruption, pathologic causes such as the development of cysts or intraosseous tumors, or due to alteration of its eruption in the presence of odontogenic tumors (1-6). In some cases, dentigerous cysts initially might have been occupying a large part of the ramus forcing and directing the molar in an altered position and then they might have been perforated and subsided (2). This theory could be in agreement with the present case.

Concerning location sites of ectopic mandibular third molars, recent literature reviews reported that in most cases they were found in the condylar region, followed by the ramus, the coronoid process, the sigmoid notch and the angulus in terms of frequency (8,9). A higher prevalence in women, during the fourth or fifth decade of life has also been reported in these reviews (8,9). However, the higher frequency reports of ectopic mandibular third molars in the condylar region may be attributed to the facts that a. their displacement in other sites may remain mainly asymptomatic and b. displacement in the condyle region may be considered more suiTable for publication than in other sites, assuming that many cases in the past concerning other heterotopic sites might have been under-reported (9).

Most usually, radiographic examination may accidentally reveal an ectopic impacted third molar when no other symptoms have been reported so far (1). Panoramic X-ray followed by a CBCT are the two radiographic techniques of choice to reveal an ectopic third molar with a concomitant pathology, such as a cystic lesion (1). There has been proposed a new classification of ectopic impacted third molars based solely on panoramic X-ray findings in attempt to serve as a new start point for optimizing clinical diagnosis and treatment (1). However, before any surgical attempt is made for ectopic impacted third molar removal, performing a CBCT is of paramount importance for the following reasons: a. it will clarify whether an intra or extraoral surgical approach is more suitable, b. it will provide the best planning of surgical field and c. it will help to determine strategies for protection of critical anatomic structures intraoperatively, thus minimizing postoperative complications (9-12).

The choice of surgical approach is based on the preference and the experience of the surgeon and the position of the ectopic tooth. In general, classical intraoral and extraoral approaches are the main two surgical techniques of choice (1,2,9). Endoscopy has also been described, since it provides magnification of the surgical field which is an important aid, especially during intraoral approaches (2). However, the cost of endoscopes and the lack of training facilities prohibit the routine use of this technique (2).

Intraoral removal of ectopic impacted third molars can be performed through the sublingual fossa or through the vestibule, depending on tooth displacement, however sagittal split osteotomy can also be performed if necessary (12). The intraoral approach is a more conservative technique and should be preferred to extraoral approach whenever possible, to avoid skin tissue scaring and potential injury of the marginal mandibular brach of the facial nerve (9-14). There are several instances, though, where the classical intraoral approach is inadequate for tooth extraction, mainly due to poor visualisation of distant regions, or in cases with trismus (10-14). Conventional intraoral approach was ruled out in the present case due to restricted surgical field and the extensive osteotomy that should have been performed, posing risks for iatrogenic mandibular fracture and inferior alveolar nerve injury.

Extraoral surgical removal is ineviTable in some cases, especially when ectopic third molars are deeply impacted/projected. However, this technique is not commonly used due to the rarity of such impactions and due to the extent of technical difficulties and associated complications, such as nerve and vascular injuries, joint damage and skin scaring (9-14). Extraoral approach for the removal of ectopic impacted third molars is indicated in cases where the molar a. is located in the condylar, sub-condylar region, ascending ramus or coronoid process, b. is located in the lower border of the mandible or in close proximity to mandibular canal, c. is classified as TMC III category (Third Molar Classification III), meaning that it is located below the mandibular canal, d. is deeply impacted and the root formation is dilacerated or hypercementosed, or when e. the patient has limited mouth opening due to severe trismus or when f. there are associated pathologies like cysts and tumours and excessive bone removal is anticipated (9,13).

The preauricular, the submandibular and retromandibular surgical approaches constitute the three different routes for extraoral access to ectopic third molars (9-13). Last two approaches are most frequently used. Choosing the most suiTable extra oral surgical approach is a matter of careful preoperative planning considering which technique is most conservative with the least possible trauma to the patient, based on the position of the ectopic tooth (8,9,13). The preauricular approach provides better access when the impaction is located in the condyle (9). The submandibular and retromandibular approaches provide better visibility if the tooth is displaced in the body and ramus of the mandible accordingly, presenting low rates of nerve injury, while the postoperative scar can be hidden in a neck wrinkle (9). In the present case, a submandibular incision was chosen due to the fact that the impaction was located in the lingual side of the lower border of the mandible.

It is always important to consider the complications that might occur from the existence and removal of ectopic mandibular third molars. Complications like jaw fracture, with or without nerve injury, might occur when excessive osteotomy is attempted and when there is limited visibility (9,14). In cases where excessive osteotomy is performed, the application of a miniplate is considered to be a higly effective preventive solution for jaw fracture (9).

In conclusion, ectopic impacted mandibular third molars represent a rare clinical entity and usually remain undiscovered unless they cause symptoms or accidentally found in a radiographic examination. In many cases they are associated with a dentigerous cyst. They can remain untreated but in cases where clinical signs of inflammation are apparent, surgical removal must be considered. Surgical approach must be carefully planned, choosing the more conservative and less traumatic technique based on tooth location. Postoperative follow-up with radiographic examination at regular intervals is mandatory.
